# Genome-wide association studies detects candidate genes for wool traits by re-sequencing in Chinese fine-wool sheep

**DOI:** 10.1186/s12864-021-07399-3

**Published:** 2021-02-18

**Authors:** Hongchang Zhao, Tingting Guo, Zengkui Lu, Jianbin Liu, Shaohua Zhu, Guoyan Qiao, Mei Han, Chao Yuan, Tianxiang Wang, Fanwen Li, Yajun Zhang, Fujun Hou, Yaojing Yue, Bohui Yang

**Affiliations:** 1 Lanzhou Institute of Husbandry and Pharmaceutical Sciences, Chinese Academy of Agricultural Sciences, Sheep Breeding Engineering Technology Research Center of Chinese Academy of Agricultural Sciences, Lanzhou, 730050 China; 2 Gansu Provincial Sheep Breeding Technology Extension Station, Sunan, 734031 China; 3 Xinjiang Gongnaisi Breeding Sheep Farm, Xinyuan, 835808 China; 4 Aohan Banner Breeding Sheep Farm, Chifeng, 024300 China

**Keywords:** Fine-wool sheep, Re-sequencing, GWAS, Enrichment analyses, Wool traits

## Abstract

**Background:**

The quality and yield of wool determine the economic value of the fine-wool sheep. Therefore, discovering markers or genes relevant to wool traits is the cornerstone for the breeding of fine-wool sheep. In this study, we used the Illumina HiSeq X Ten platform to re-sequence 460 sheep belonging to four different fine-wool sheep breeds, namely, Alpine Merino sheep (AMS), Chinese Merino sheep (CMS), Aohan fine-wool sheep (AHS) and Qinghai fine-wool sheep (QHS). Eight wool traits, including fiber diameter (FD), fiber diameter coefficient of variance (FDCV), fiber diameter standard deviation (FDSD), staple length (SL), greasy fleece weight (GFW), clean wool rate (CWR), staple strength (SS) and staple elongation (SE) were examined. A genome-wide association study (GWAS) was performed to detect the candidate genes for the eight wool traits.

**Results:**

A total of 8.222 Tb of raw data was generated, with an average of approximately 8.59X sequencing depth. After quality control, 12,561,225 SNPs were available for analysis. And a total of 57 genome-wide significant SNPs and 30 candidate genes were detected for the desired wool traits. Among them, 7 SNPs and 6 genes are related to wool fineness indicators (FD, FDCV and FDSD), 10 SNPs and 7 genes are related to staple length, 13 SNPs and 7 genes are related to wool production indicators (GFW and CWR), 27 SNPs and 10 genes associated with staple elongation. Among these candidate genes, *UBE2E3* and *RHPN2* associated with fiber diameter, were found to play an important role in keratinocyte differentiation and cell proliferation. Gene ontology (GO) and Kyoto Encyclopedia of Genes and Genomes (KEGG) enrichment results, revealed that multitude significant pathways are related to keratin and cell proliferation and differentiation, such as positive regulation of canonical Wnt signaling pathway (GO:0090263).

**Conclusion:**

This is the first GWAS on the wool traits by using re-sequencing data in Chinese fine-wool sheep. The newly detected significant SNPs in this study can be used in genome-selective breeding for the fine-wool sheep. And the new candidate genes would provide a good theoretical basis for the fine-wool sheep breeding.

**Supplementary Information:**

The online version contains supplementary material available at 10.1186/s12864-021-07399-3.

## Background

The wool industry produces approximately 1160 million kilograms of clean wool every year from a global herd of over a billion sheep. The economic value of wool depends on various parameters such as the fiber diameter, fleece weight, clean fleece rate and staple strength. In general, wool traits are affected by diverse genetic and environmental factors simultaneously, with moderate to low heritability [1]. For a fine-wool sheep breeder, understanding the genetic background and detecting genetic markers associated with wool traits can facilitate improved genetic selection for desirable traits to accelerate the genetic progress. Biologically, the growth process of wool is related to the wool follicle development [2, 3], wool follicle growth cycle [4, 5], and hair follicle stem cell differentiation [6–8]. These processes involve complex coordination among various genes and cell types, and occurs in the skin [9]. Mutations in related genes and status changes in the corresponding cells potentially affects the wool traits. From the perspective of genetic control, the detection of candidate genes associated with wool traits is particularly important. Furthermore, in fine-wool sheep breeding, measuring the wool phenotype data is complex and expensive [10]. Therefore, genomic approaches are an essential step for the fine-wool sheep breeding.

With the development of sequencing technology and commercial SNP array genotyping technologies, researchers can now identify quantitative trait loci (QTL) by performing genome-wide association studies (GWAS) between genetic markers and phenotypic records [11]. GWAS offers advantages in detecting narrow genomic regions of causal variants with a modest impact on important economic traits and can hence be regarded as the first step toward to understand the molecular and cellular mechanisms underlying the phenotypic expression of complex traits [12]. GWAS has been successfully implemented in mapping QTL for economically important traits in the livestock breeding populations [13, 14]. In sheep breeding, the genetic mechanism behind economically complex traits is generally complex and controlled by multiple genes. GWAS have been conducted to detect genetic variants for economic traits in sheep [15, 16], and several studies have reported the presence of candidate genes for wool traits in a variety of sheep breeds. Moreover, genome-wide significant SNPs associated with wool traits in Chinese Merino Sheep (JunKen type) and yearling wool traits in Chinese Merino sheep have been detected by using OvineSNP50k BeadChip [10, 17]. In addition, single-trait GWAS, multi-trait GWAS, and identified putative QTL for wool traits have been conducted in both Merino and Merino crossbred sheep by using OvineHD BeadChip [18]. These studies have provided several beneficial genetic markers for fine-wool sheep breeding.

However, genotype data of the above mentioned studies were obtained based on the SNP array. The currently available commercial SNP array such as the Illumina Ovine SNP50K BeadChip cannot cover all the SNPs involved in the fine-wool sheep genome. Given to the limited number of SNPs, the power of GWAS is also limited, indicating that some genes affecting traits may not be detected. Eventually this may cause difficulty in understanding the molecular mechanisms of wool trait formation. Whole genome re-sequence data containing the majority of SNPs were optimized to enhance the accuracy and power of GWAS. With reference to the genetic background of Chinese fine-wool sheep breeds, the previous GWAS was mainly based on one breed, which inevitably affected the applicability of QTL for wool traits. In this study, we utilized the re-sequencing data and wool phenotypic data of 460 sheep belonging to four different genetic backgrounds of fine-wool sheep breeds in China including Alpine Merino sheep (AMS), Chinese Merino sheep (CMS), Aohan fine-wool sheep (AHS) and Qinghai fine-wool sheep (QHS) to conduct GWAS aiming to explore the candidate genes and the common potential causal genetic variants involved in the development of wool traits in different breeds. We thus expect that the potential genetic markers identified in this study will be applicable to genome-selective breeding of fine-wool sheep across the world. Moreover, we believe that the detected candidate genes will facilitate the comprehension of the development mechanisms of wool traits in the future.

## Results

### Summary statistics of phenotype data and sequencing data

The descriptive statistics of eight phenotypic wool traits and the numbers of sheep are presented in Table 1 and Supplementary Table S1. The phenotypic values are approximately normally distributed by using the 3σmethod. The sequencing step generated 8.222 Tb of raw data, with an average 17.874Gb of raw data for each sample, while 8.190 Tb of filtered clean data was obtained, with an average of 17.803Gb data for each sample (Supplementary Table S2). The sequencing quality was high with an average Q20 of 97.71% and an average Q30 of 92.34%. The distribution of GC content in the 460 samples ranged from 41.58 to 47.31%, indicating successful library construction and sequencing. Based on our mapping results (Supplementary Table S3), the average mapping rate reached 99.01%, with the highest rate at 99.41% and the lowest at 97.44%. In alignment with the reference sequence, the average coverage depth was 8.59X. Following filtration and screening, 12,561,225 SNP sites met the requirements of genome-wide resequencing, and the SNP density plot of each chromosome is illustrated in Supplementary Fig. S1.

**Table 1:** Descriptive statistics for the wool traits evaluated

Wool traits (unit)	Mean ± SD	Minimum	Maximum	No. individuals
FD (μm)	20.87 ± 2.00	16.2	26	460
FDCV (%)	19.21 ± 2.86	12.7	27.8	459
FDSD (μm)	3.99 ± 0.66	2.5	6	456
SL (cm)	9.68 ± 1.15	5	13	385
GFW (kg)	6.72 ± 2.64	2.99	12.8	428
CWR (%)	58.61 ± 8.98	31.17	76.15	458
SS (N/ktex)	30.97 ± 8.59	7.39	53.64	460
SE (%)	21.94 ± 6.55	8.54	45.01	453

FD mean fibre diameter, *FDCV* fibre diameter coefficient of variation, *FDSD* fibre diameter standard deviation, *SL* staple length, *GFW* greasy fleece weight, *CWR* clean wool rate, *SS* staple strength, *SE* staple elong;

### Principal component and LD analysis

**Fig. 1:**
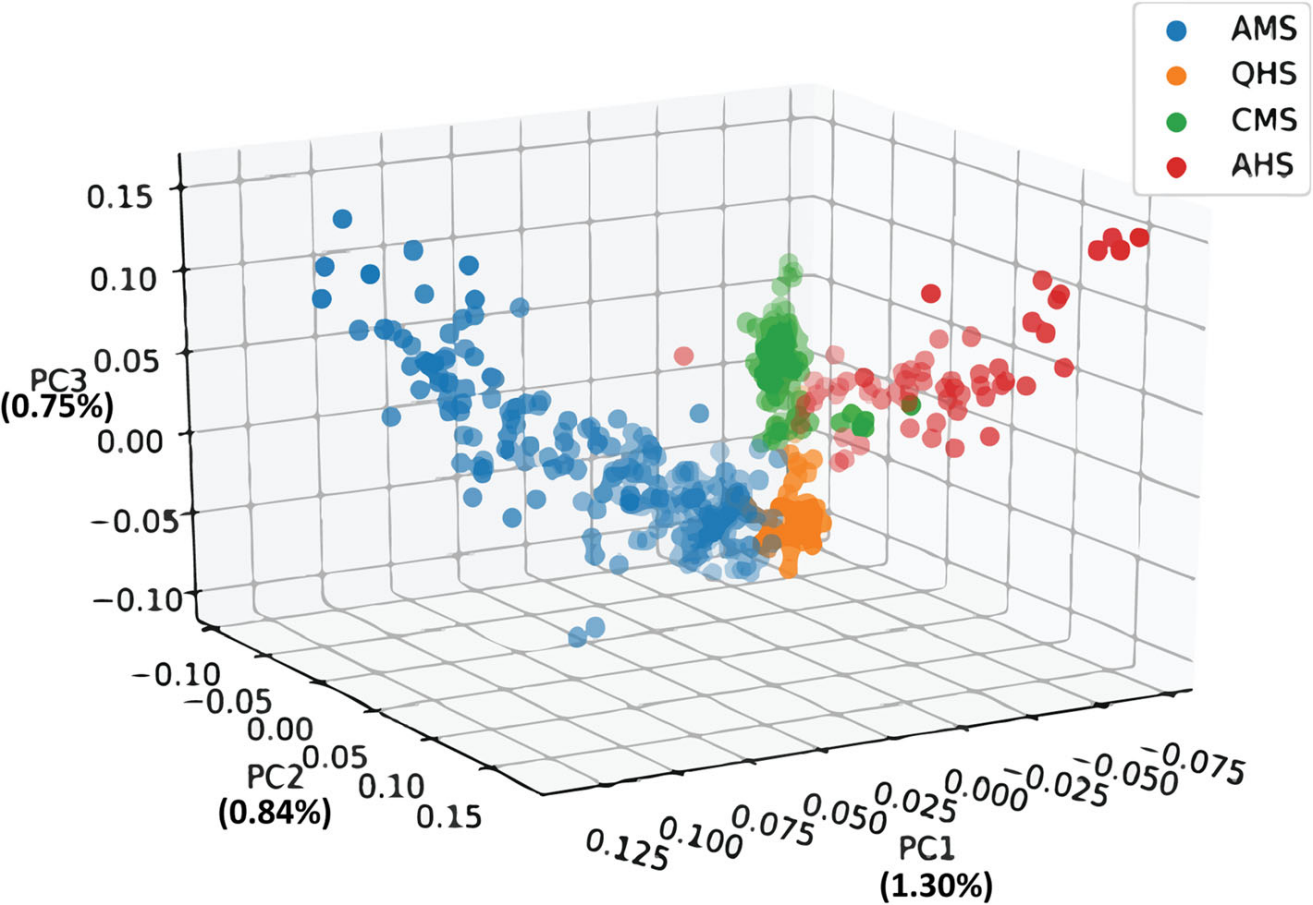
PCA analysis for the four fine-wool sheep

The population stratification revealed different genetic backgrounds contributed by factors such as different varieties, strains, and family. The GCTA software was used to conduct PCA on the AMS, CMS, AHS, and QHS population in order to understand their genetic background. The AMS was more dispersed than the CMS group, based on the first two principal component. With regard to the composition and the second principal component, the AHS group was more dispersed than the CMS group. However, the QHS group was not separated from the other groups. In fact, the genetic background of the CMS, AHS, and AMS showed some differences, albeit they were not completely separated. The scatterplots of the first (1.30%), second (0.84%), and third (0.75%) principal components are displayed in Fig. 1.

The linkage disequilibrium (LD) decay is illustrated in Supplementary Fig. S2. With increasing distance in the AMS population, the LD dropped more quickly than in the other three breeds. For SNPs up to 50 kb apart, the average r^2^ values were equal to 0.056 (AMS), 0.077 (AHS), 0.073 (CMS), 0.066 (QHS), and 0.045 (Total). Further details about the LD analysis using r^2^ are included in Supplementary Table S4. Our results indicated that the LD decay tends to be stable when the distance is 100 kb. Therefore, we considered the genes located within ±50 kb near the significant SNP sites as the candidate genes.

### Estimation of genetic parameters

Genetic variance, residual variance, and the heritability of wool traits were estimated by the AI-REML using the genomic BLUP (gBLUP) for the data of four breeds. The estimated genetic parameters of wool traits are shown in Table 2. The estimated heritability of wool traits was 0.44–0.77. Among them, the highest heritability was observed for GFW (0.77) and the lowest for SL (0.44). The estimated heritabilities of FD, FDSD, and FDCV were 0.64, 0.65, and 0.45, respectively, which indicated high heritability traits.

**Table 2:** Estimation of genetic parameters of eight wool traits

Trait (unit)	Additive genetic variance ±SE	Residual variance ± SE	h^2^ ± SE
FD (μm)	1.95 ± 0.45	1.11 ± 0.40	0.64 ± 0.13
FDCV (%)	2.66 ± 1.20	3.27 ± 1.13	0.45 ± 0.19
FDSD (μm)	0.21 ± 0.06	0.11 ± 0.05	0.65 ± 0.17
SL (cm)	0.51 ± 0.25	0.65 ± 0.23	0.44 ± 0.20
GFW (kg)	1.75 ± 0.30	0.53 ± 0.25	0.77 ± 0.11
CWR (%)	21.81 ± 5.77	14.50 ± 5.20	0.60 ± 0.15
SS (N/ktex)	31.53 ± 10.74	22.43 ± 9.77	0.58 ± 0.19
SE (%)	22.44 ± 6.48	13.77 ± 5.83	0.62 ± 0.16

FD mean fibre diameter, *FDCV* fibre diameter coefficient of variation, *FDSD* fibre diameter standard deviation, *SL* staple length, *GFW* greasy fleece weight, *CWR* clean wool rate, *SS* staple strength, *SE* staple elong;

### Wool traits genome-wide association studies

**Fig. 2:**
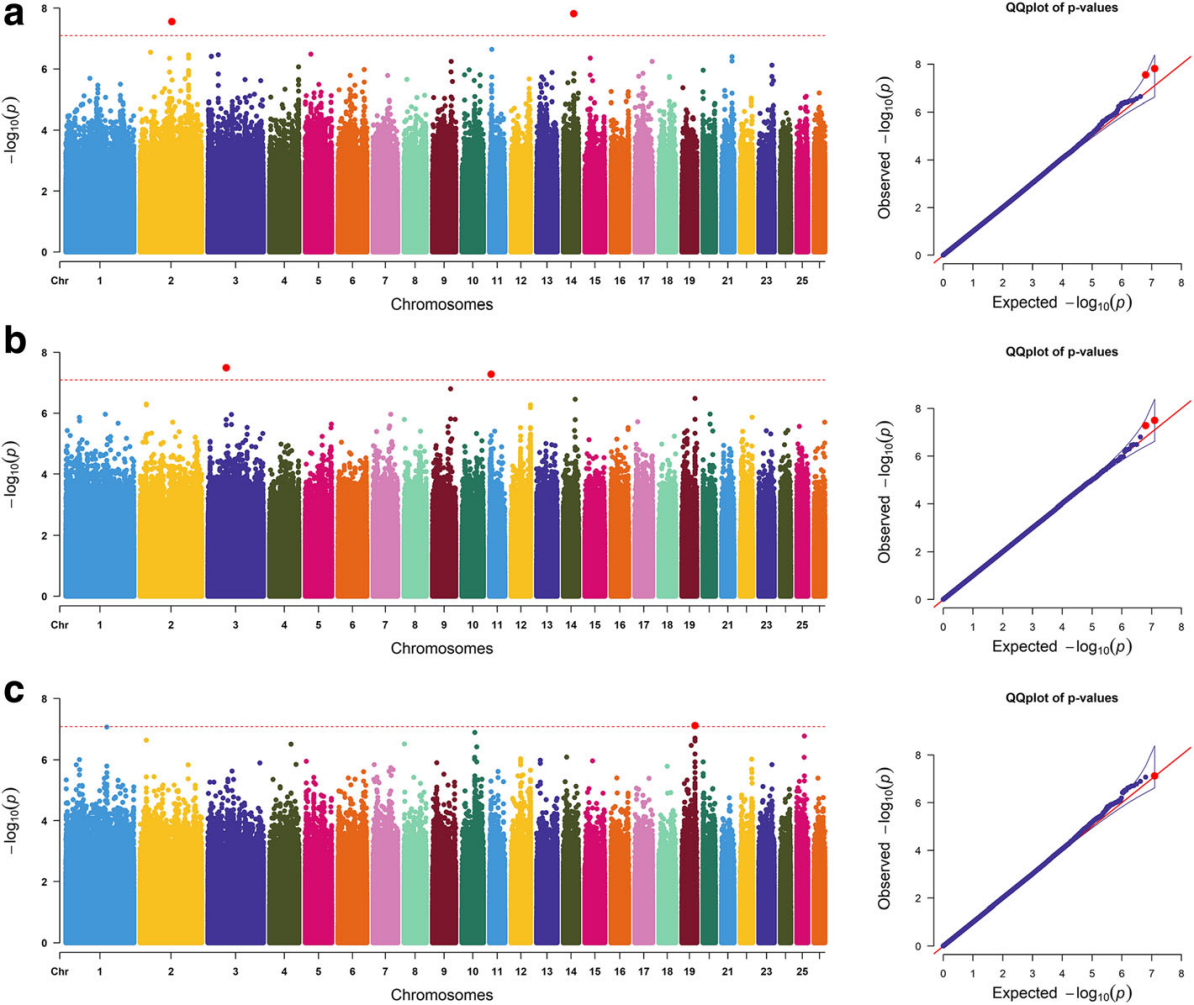
Manhattan plots and QQ plots of **a** FD, **b** FDCV, and **c** FDSD traits. The point of significant SNPs are depicted in red color

Using the general linear model, we found that the sheep sex had a significant influence on the resultant phenotypic values. Therefore, we added sex information as a fixed effect to the mixed linear model. We detected 57 significant associated SNP loci at the genome level, the detailed information about the significant SNPs is displayed in Table 3. After gene annotation, 30 candidate genes were finally identified as being related to wool traits. In addition, 10 genes were not officially named, but were represented by their location information. For instance, *LOC101117971* was related to FDSD. For the FD trait, 2 significant SNPs were detected on OAR2 (OAR: *Ovis aries* chromosome) and OAR14. The most significant SNP annotated *RHPN2* was located on OAR14 (Fig. 2a). Two significantly correlated SNPs were detected for the FDCV trait, and the candidate regions were located on the OAR3 and OAR11. The most significant SNP within *NRXN1* was located on OAR14 (Fig. 2b). Three significant SNPs were detected for the FDSD trait, and the candidate regions were located on the OAR12 and OAR19. The most significant SNP within *LOC101117971* was located on OAR19. Two loci were identified on OAR19 in the *LOC101117971*, and these 2 loci were only 25-bp apart (Fig. 2c).

**Table 3:** Significant SNPs associated with seven wool traits

Trait	Chr	position (bp)	*P*-value	Candidate genes	Distance (bp)
FD	2	127,805,728	2.80E-08	***UBE2E3***	within
	14	42,678,119	1.52E-08	***RHPN2***	4985
FDCV	3	74,006,775	3.19E-08	***NRXN1***	within
	11	6,520,617	5.25E-08	***ANKFN1***	within
FDSD	12	38,844,498	6.60E-08	***TNFSF4***	−38,182
	19	53,142,776	2.31E-08	***LOC101117971***	within
	19	53,142,801	3.23E-08	***LOC101117971***	within
SL	1	211,531,257	2.35E-08	***NLGN1***	within
	1	205,410,405	3.64E-08	***USP13***	within
	4	50,182,043	8.08E-09	***LOC101108907***	18,447
	9	82,560,062	1.09E-09	***GEM***	760
	9	82,565,963	3.11E-08	***GEM***	6661
	9	82,560,051	3.28E-08	***GEM***	749
	9	82,565,750	4.22E-08	***GEM***	6448
	17	68,092,867	1.02E-08	***EWSR1***	within
	17	68,313,142	1.37E-08	***NF2***	within
	24	29,022,455	2.74E-08	***CALN1***	16,070
GFW	2	89,156,912	7.96E-09	***LOC101103702***	within
	2	20,077,571	9.50E-09	***–***	
	2	20,343,753	1.64E-08	***–***	
	2	22,673,981	7.32E-08	***LOC101123603***	−32,038
	3	9,591,330	1.63E-11	***MVB12B***	within
	3	9,591,125	3.41E-08	***MVB12B***	within
	3	84,111,139	6.05E-08	***–***	
	11	48,617,494	6.61E-08	***PITPNC1***	within
	24	30,292,869	4.70E-08	***LOC105604756***	within
	25	37,386,229	1.56E-08	***NRG3***	within
CWR	1	78,731,004	4.92E-08	***LOC101117031***	within
	8	50,472,213	5.28E-08	***–***	
	10	32,755,294	1.44E-08	***–***	
SS	1	96,961,763	7.87E-12	***LOC101112943***	within
	1	84,208,941	4.05E-09	***VAV3***	within
	2	182,741,643	1.01E-08	***LOC105610635***	within
	2	180,636,507	1.11E-08	***–***	
	3	108,977,664	1.55E-08	***–***	
	3	108,977,663	2.31E-08	***–***	
	3	108,977,665	2.79E-08	***–***	
	7	57,497,429	4.6E-08	***LOC105607291***	within
	7	57,544,088	6.95E-08	***LOC105607291***	within
	12	65,659,038	5.67E-10	***–***	
	12	65,676,686	8.32E-10	***–***	
	12	65,676,784	2.76E-09	***–***	
	12	65,666,086	3.99E-09	***–***	
	12	65,665,315	7.77E-08	***–***	
	13	65,826,724	5.95E-08	***SRC***	28,588
	15	21,954,509	6.51E-10	***BCO2***	within
	15	51,815,926	3.38E-08	***PGM2L1***	within
	15	51,845,450	3.71E-08	***PGM2L1***	within
	15	21,953,940	5.87E-08	***BCO2***	within
	15	21,955,321	6.14E-08	***BCO2***	within
	15	51,810,577	6.29E-08	***PGM2L1***	within
	18	59,961,387	1.41E-08	***PAPOLA***	− 6293
	18	36,751,894	4.77E-08	***–***	82,558
	18	36,747,715	7.16E-08	***–***	
	18	36,789,189	7.83E-08	***–***	
	22	37,530,124	1.16E-08	***FAM204A***	12,749
	22	21,182,419	6.28E-08	***LOC105604253***	15,932

FD mean fibre diameter, *FDCV* fibre diameter coefficient of variation, *FDSD* fibre diameter standard deviation, *SL* staple length, *GFW* greasy fleece weight, *CWR* clean wool rate, *SS* staple strength

**Fig. 3:**
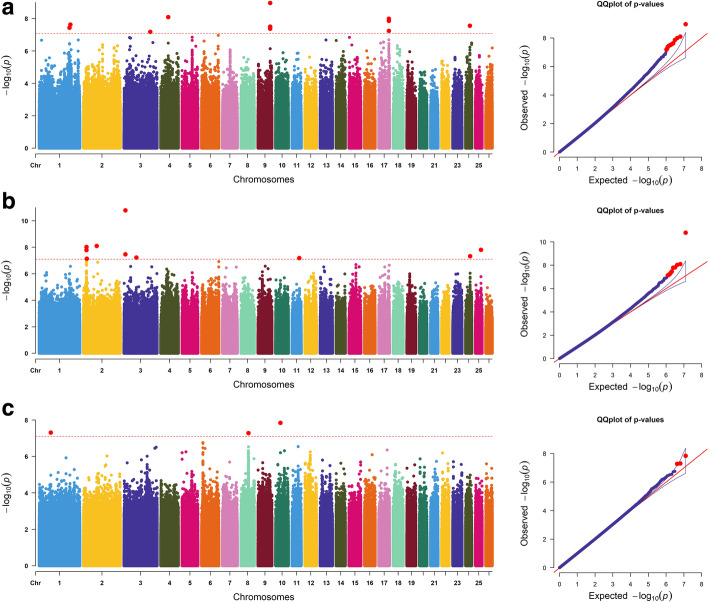
Manhattan plots and QQ plots of the **a** SL, **b** GFW and **c** CWR traits. The points of significant SNPs are depicted in red color

For the SL trait, 10 significantly correlated SNPs were detected on OAR1, 4, 9, 17, and 24. Among them, the most significant SNP within *EWSR1* was located on OAR17. Four loci were annotated to the same gene *GEM* on OAR9. (Fig. 3a). For the GFW trait, 10 significant SNPs were detected on the OAR2, 3, 11, 24, and 25. Among these SNPs, the most significant SNP unannotated was located on chromosome 14. Two sites on OAR2 were not annotated to the gene, 2 sites on OAR3 were in *MVB12B*, but one site was not annotated to the gene (Fig. 3b). Three significantly correlated SNPs were detected for the CWR trait, and the candidate regions were located on OAR1, 8 and 10. Among them, the loci of chromosomes 8 and 10 were not annotated to genes (Fig. 3c).

**Fig. 4:**
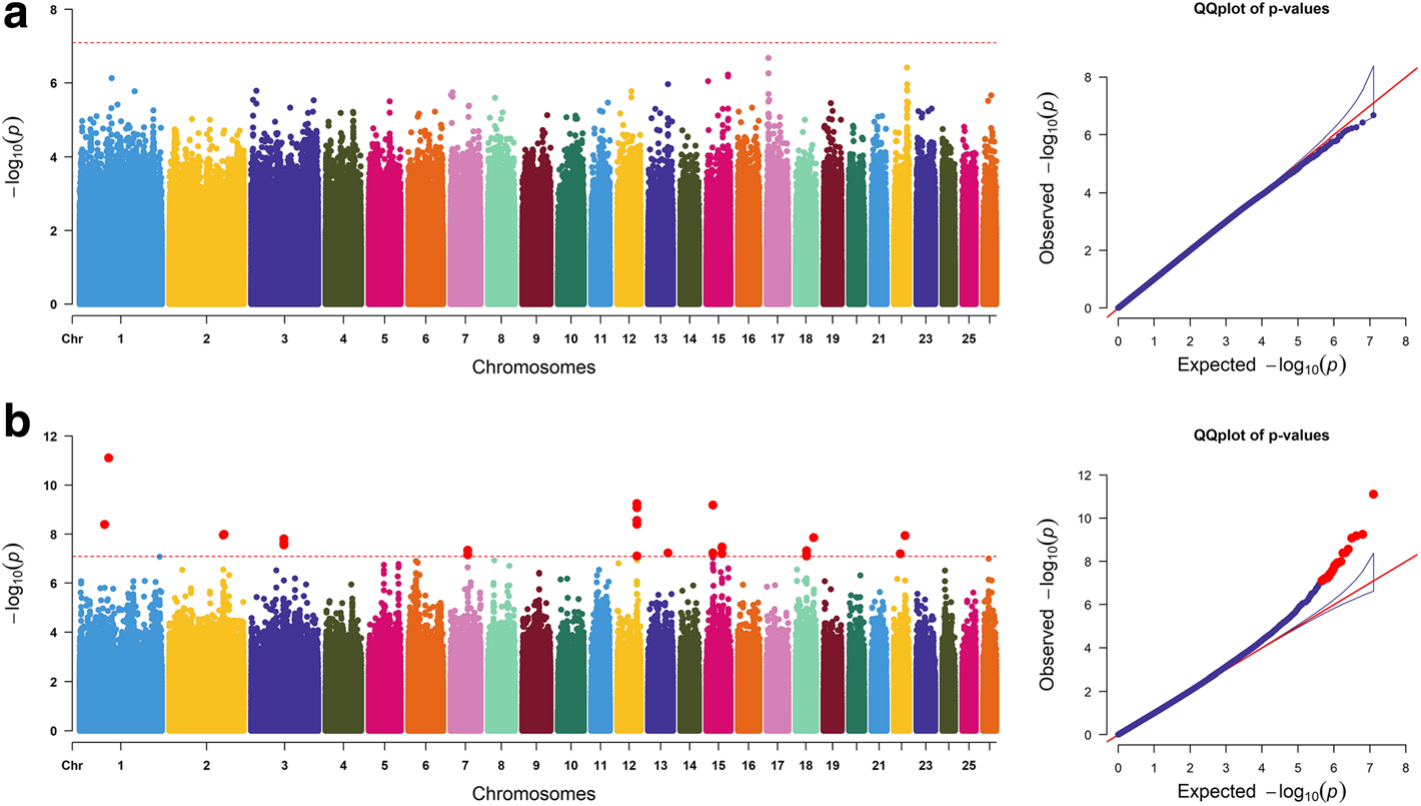
Manhattan plots and quantile-quantile (QQ) plots of both the **a** SS and **b** SE traits. The points of genome-wide significant SNPs are depicted in red color. No points were detected in the SS traits

For the SE trait, 27 significantly correlated SNPs were detected on OAR1, 2, 3, 7, 12, 13, 15, 18, and 22. Among them, the most significant SNP in *LOC105610635* was located on OAR19; three loci on OAR15 were located in *PGM2L1*, and three loci were located on *BCO2*. In addition, three loci on OAR18 were not annotated to the gene (Fig. 4b). However, no SNPs surpassed the genome-wide significance threshold for the SS trait.

### Enrichment analysis

To evaluate the characteristics of the candidate genes in detail annotated by significant SNPs, we enriched these genes further. We performed the enrichment analysis on these genes annotated at the SNP sites with *p*-value<2E-06 (Supplementary Table S5) related to the above wool traits by GO and KEGG. Among them, FD, FDCV and FDSD were put together for enrichment analysis. The biological processes in GO (Table 4) that maintained the statistical significance included the integral component of the endoplasmic reticulum (ER) membrane (*p* = 6.72E-05), positive regulation of monocyte chemotaxis (*p* = 0.02651675), and presynaptic membrane (*p* = 0.030568552). The p-value of the GO term associated with SL and GFW was approximately 0.05. Among them, *WDPCP* and *KANK1* were enriched in the regulation of cell polarity establishment. *PID1* was enriched in the negative regulation of insulin receptor signaling pathway (GO:0046627). In terms of the wool stretch performance, the most significant pathway showed a positive regulation of canonical Wnt signaling (*p* = 0.01). There are two pathways related to the bone, osteoclast development (p = 0.01), and bone resorption (*p* = 0.04), namely, the extracellular-glutamate-gated ion channel activity (*p* = 0.039331749) and the excitatory postsynaptic potential (*p* = 0.051115614). The KEGG pathway analysis revealed that the candidate genes were significantly enriched in the four pathways (Table 4). We identified that the pathways related to FD involved cytokine-cytokine receptor interaction, which in turn involved four candidate genes *LOC101103766*, *TNFSF4*, *LOC101117971*, and *TNFSF18* as well as glycosaminoglycan biosynthesis for heparan sulfate and heparin.

**Table 4:** Results of enrichment by GO and KEGG analysis for the candidate genes

Trait	Term	*P*-value	Enrich Genes
FD, FDCV and FDSD	integral component of endoplasmic reticulum membrane (GO:0030176)	0.0000672	*SAMD8, FKBP8, EXT1, DCSTAMP*
	positive regulation of monocyte chemotaxis (GO:0090026)	0.02651675	*LOC101117971, TNFSF18*
	presynaptic membrane (GO:0042734)	0.030568552	*CADPS2, NRXN1*
	positive regulation of inflammatory response (GO0050729)	0.052361511	*TNFSF4, TNFSF18*
	Cytokine-cytokine receptor interaction (oas04060)	0.012537021	*LOC101103766, TNFSF4, LOC101117971, TNFSF18*
	Glycosaminoglycan biosynthesis - heparan sulfate heparin (oas00534)	0.053641685	*HS6ST3, EXT1*
SL and GFW	regulation of establishment of cell polarity (GO:2000114)	0.05354365	*WDPCP, KANK1*
	negative regulation of insulin receptor signaling pathway (GO:0046627)	0.057544264	*PID1, KANK1*
	nucleotide binding (GO:0000166)	0.074031682	*LARP6, ADCY1, EIF3B, EWSR1*
	regulation of autophagy (GO:0010506)	0.081203017	*ABL2, USP13*
	Cell adhesion molecules (CAMs) (oas04514)	0.009109937	*LOC101108158, CADM1, NLGN1, CNTNAP2*
	Glutamatergic synapse (oas04724)	0.031888822	*ADCY1, GRIA2, GRM7*
SS and SE	positive regulation of canonical Wnt signaling pathway (GO:0090263)	0.010663274	*YAP1, LRRK1, SRC*
	osteoclast development (GO:0036035)	0.011584332	*LRRK1, SRC*
	extracellular-glutamate-gated ion channel activity (GO:0005234)	0.039331749	*GRIN2A, GRID2*
	bone resorption (GO:0045453)	0.042775594	*LRRK1, SRC*
	excitatory postsynaptic potential (GO:0060079)	0.051115614	*GRIN2A, GRID2*

FD mean fibre diameter, *FDCV* fibre diameter coefficient of variation, *FDSD* fibre diameter standard deviation, *SL* staple length, *GFW* greasy fleece weight, *SS* staple strength, *SE* staple elong;

## Discussion

Generally, the development of wool traits involves a complex genetic mechanism. Therefore, identifying the genes underlying these traits is essential for fine-wool sheep breeding. With the advent of SNP genotyping technologies, GWAS has become feasible, and has been performed in the ovine species to investigate wool traits [10, 18]. However, these studies were based on SNP array, and may missed some key SNPs inevitably involved with wool traits. In this study, we re-sequenced samples from four fine-wool sheep breeds (AMS, CMS, AHS, and QHS) and then we performed GWAS to detect candidate genes that influence eight beneficial wool traits. In this study, the SNP quantity increased by 12 M.

The statistical power to detect the associations in GWAS mainly depended on the LD between markers and QTL. In our analysis, AHS, CMS, and QHS showed a higher average r^2^ values in comparison to AMS, and the average r^2^ value was the lowest when all breeds were mixed together. As is known, AMS is a newly cultivated breed with greater genetic diversity than the earlier breeds. Our results indicate that the LD decay was breed-specific, which conforms to the reports of previous research [19].

The most important role of heritability is to estimate the reliability of phenotypic values as breeding values [20]. The heritability value of each trait is not unique, rather it is related to various factors, such as the breeds and the environment. The heritability of each wool trait in our study ranged from 0.44 to 0.77. According to the related published reports, the heritability of FD in fine-wool sheep is from 0.19 to 0.62 [21–23]. The estimated heritability of the FD traits in this study was 0.64, which is relatively close to the previously reported values. Notably, heritability of the GFW trait in our study was 0.77, which is much greater than that reported previously, such as for the Chinese superfine merino sheep the heritability of the GFW is 0.17 [23]. The reason for the difference in heritability may be attributed to several factors such as the data structure, model construction, application software, and calculation methods. In the process of calculating heritability, considering that our population does not have pedigree information, we employed the kinship established by genomic information, which may have contributed to the different results.

During the associated analysis, we considered that group stratification and individual kinship were the main factors that affected the false-positive associations. Statisticians and geneticists have developed several methods to overcome these two influencing factors [24–26]. Moreover, the EMMAX approach could use an empirical relatedness matrix which encodes a wide range of sample structure, including population stratification and hidden relatedness [27]. Therefore, we used the mixed linear model (EMMAX algorithm) for GWAS. Whether the model for each trait was appropriate could be judged by the quantile-quantile (QQ) plots. The QQ plots in our study revealed that the actual and theoretical values of most loci were consistent, which in turn indicates that the EMMAX method was effective with respect to the hierarchical population control. The wool traits values are generally affected by sex [28]. Therefore, we also added sex as a fixed factor to reduce the effect of sex on a particular phenotype.

As the Bonferroni method is extremely restrictive, some useful sites may be screened [29]. Based on our results, some genes showed values below the threshold line (−log10(*p-*values) = 7.1), in consistence with previous reports. For instance, *EXT1* located on OAR9 include the SNPs (75.5 Mb, *p* = 5.68E-07) was associated with FD near a known QTL (75.9 Mb) [10]. *PAX9* located on OAR18, which include the SNPs (45.6 Mb, *p* = 8.13E-07), was identified to be related to hair defects [30]; this finding agrees with the genome-wide selection signatures for wool traits among Merino and Merino-derived sheep breeds [31]. *KRT17* located on OAR11, which is near the SNPs (41.2 Mb, *p* = 1.34E-06), is required for the correct growth of hair follicles, especially for continuous growth [32]. Therefore, we raised the *p* = 2E-06 value as the threshold to search for more genes for the enrichment as analysis in order to better understand the functions of candidate genes.

In this study, seven SNPs were found to be associated with FD-related traits (such as FD, FDCV, and FDSD), which were then used to evaluate the characteristics of fiber diameter. More interestingly, three SNPs lying in the genomic regions, including three candidate genes (*UBE2E3*, *TNFSF4****,*** and *RHPN2*), participated in cell differentiation or proliferation. A past study verified that *UBE2E3* is required for cell proliferation [33]. In our study, the mitotic activity of germ cells in the hair follicles were found to affect the cell content of wool fibers, which resulted in the development of a variety of fiber diameter. *TNFSF4* can regulate apoptosis, proliferation, and differentiation of cells [34], as well as enriched into the cytokine-cytokine receptor interaction (oas04060) in our KEGG results. Keratinocytes are known to produce and release a series of cytokines that contribute to cell growth and differentiation [35]. At the transcription level, a previous experiment revealed that the *TNFSF4* expression is significant for the outer root sheath (ORS) of postnatal mouse skin [36]. In addition, a previously research demonstrated that *TNFSF4* plays a role in the feather follicle initiation as well as in the development phase of feather follicle morphogenesis [37]. The SNPs (OAR12:38.8 Mb) annotated *TNFSF4* were located next to the genomic regions by selection signature detection in Spanish Merino [31]. Some genes indirectly control the cell differentiation. *RHPN2* is involved in the Rho pathway [38]. Although more critical, keratinocyte differentiation is regulated by the Rho signaling pathway [39]. In addition, a previous study has shown that *RHPN2* was upregulated in the production of hair follicle stem cells in mice [40].

For another important wool trait SL, we obtained 10 significantly associated SNPs. Although these sites were not located in the known QTL, three candidate genes (namely, *NLGN1*, *GEM*, and *NF2*) were related to cell differentiation or proliferation. From the perspective of affecting cell differentiation, *NLGN1* is a cell surface protein involved in cell-cell interactions that is enriched into significant cell adhesion molecules (oas04514), which play an essential function of keratinocyte differentiation control of cell adhesion [41]. In addition, previous research has shown that the downregulation of *NLGN1*, promoted morphogenesis in the early hair follicle placodes in mice [42]. In terms of genes related to cell proliferation, *GEM* belongs to a new family within the Ras superfamily that is involved in the origination of mouse keratinocyte stem cells from the bulge of hair follicles [43]. Furthermore, previous research has shown that *GEM* may be a candidate gene for the large colonies-keratinocyte stem cell locus 2 (KsC2), which is produced in cellular responses to growth stimulation [44]. On the other hand, the gene that inhibits cell proliferation, such as *NF2* (Merlin), is a regulator of the Hippo signaling pathway that plays a key role in tumor inhibition by limiting proliferation and promoting cell apoptosis [45].

For the SE trait, the function of the candidate gene *VAV3* is a guanine nucleotide exchange factor. A previously published research reported *VAV3* only in the bulge outer root sheath (ORS) via gene expression profiling [46]. In addition, the inhibition of *VAV3* transcripts induced cytoskeleton defects, increased the cell area and enhanced the fiber stress [47]. Another associated gene *SRC* is an important component of adhesion reaction, and the cell-cell contact between neighboring keratinocytes is mainly mediated by connecting the junctions and desmosomes [48]. A previous research showed that keratinocytes differentiation could reduce the tyrosine phosphorylation activities, which play a positive role in controlling cell adhesion in the skin of mice [41]. In our study, *SRC* was enriched into the Canonical Wnt signaling (GO:0090263), which is essential for the induction of hair follicle development [49]. The Canonical Wnt signaling pathway dictated the follicle distribution [50], and is required in the normal skin to guide the differentiation of bulge stem cells into hair cells [51]. *SRC* was also enriched into the osteoclast development (GO:0036035), and the interaction between keratinocytes and fibroblasts induces osteoclasto genesis, which in turn can lead to bone resorption [52]. This process may reduce the toughness of the wool fiber, making it easily breakable.

Based on extensive search and literature review, we believe that this is the first GWAS research for wool traits in multiple Chinese fine-wool sheep breeds via re-sequencing data. Despite our results being different from those of previous studies for wool traits, the functions of candidate genes detected in our study were directly or indirectly related to keratinocytes or wool follicles development. The disagreement in our results relative to that in previous studies can be possibly attributed to the difference in the breed and the density and quantity of SNPs used in the analysis. These newly detected candidate genes may be involved in the formation of wool traits, warranting further research of the biological function of these genes.

## Conclusions

In this study, we performed genome-wide resequencing of 460 sheep belonging to four fine-wool sheep breeds (namely, AMS, CMS, AHS, and QHS). A total of 8.222 Tb of raw data was generated, with an average of approximately 8.59X sequencing depth. After filtration and screening, 12,561,225 SNPs yielded. GWAS was conducted for eight wool traits by using the EMMAX model. A total of 57 genome-wide significant SNPs were detected in associated with the selected wool traits. Moreover, 30 candidate genes were detected within the 100-kb area near the genome-wide significant SNPs. Among them, *UBE2E3* and *RHPN2* are known to play important roles during keratinocyte differentiation and cell proliferation. Most of the significant GO and KEGG pathways were found to be related to keratin and cell proliferation as well as to differentiation. The newly detected significant SNPs in this study can be applied to genome-selective breeding for fine-wool sheep. The new candidate genes are expected to provide a good theoretical basis for optimizing fine-wool sheep breeding.

## Methods

### Phenotypes and re-sequence data

Wool traits were sampled from 460 sheep (adults aged > 550 days) belonging to 4 fine-wool sheep breeds in China. Briefly, 220 AMS (75 male, 145 female sheep) were sampled from Gansu Provincial Sheep Breeding Technology Extension Station (Huangcheng, Gansu, China); 120 CMS (60 male, 60 female sheep) were sampled from Gongnaisi Breeding Sheep Farm (Gonnaisi, Xinjiang Uygur Autonomous Region, China); 60 AHS (30 male, 30 female sheep) were sampled from Aohan Banner Breeding Sheep Farm (Chifeng, Inner Monglolia Autonomous Region, China) and 60 QHS (30 male, 30 female sheep) were sampled from Sanjiaocheng Sheep Farm (Sanjiaocheng, Qinghai, China). All sheep in this study were randomly selected without considering any pedigree information.

The wool samples were collected from the posterior edge of the sheep’s scapula, slightly above the midline of the sheep’s body. We released the sheep after sampling in our study. The samples were dispatched to the National Animal and Rural Ministry of Animal and Fur Quality Supervision and Inspection Center (Lanzhou, China), where the following seven wool traits were measured and recorded. Fiber diameter (GB/T 10685–2007), FD coefficient of variance (GB/T 10685–2007), FD standard deviation (GB/T 10685–2007), staple length (GB/T 6976–2007), clean fleece rate (GB/T 6978–2007), staple strength (GB/T 13835.5–2009), and staple elongation (GB/T 13835.5–2009). The operating procedures were in accordance with the corresponding national standards. The greasy fleece weight trait was measured on-site at the sampling farm.

### Sequence alignment, quality control, population SNP detection, and SNP annotation

Genomic DNA was extracted by the phenol-chloroform method using sheep whole blood (5 mL) collected through the jugular vein into vacutainers containing anticoagulant K_2_EDTA [53]. We released all the sheep after blood collection in this study. All samples used for genome sequencing were processed on the Illumina HiSeq Xten platform. The resulting data was considered as valid data. For filtering the raw data, the below-mentioned method was followed:

1. Reads containing the linker sequence were filtered out;
2. The N content in single-ended access reads exceeding 10% of the read length was set as the standard for deleting paired reads;
3. When the number of low-quality (≤5) bases contained in the single-ended sequencing read exceeded 50% of the length of the read length, the paired reads were removed.

High-quality sequencing data were aligned to the reference sheep genome assembly Oar_v4.0 using the Burrows-Wheeler Aligner (BWA) software [54] (Parameter: mem-t 4-K 32-M). Duplicates were removed by using SAMtools [55] (parameter: rmdup). Finally, the sample comparison rate was statistically analyzed.

We used the SAMtools [55] to detect SNPs in the population samples and to obtain high-quality SNPs through the following filtering and screening approach: i) the support number (coverage depth) of SNP was >2; ii) the proportion of MIS (missing) was <10%; and iii) minimum allele frequency (MAF) was >5%. ANNOVAR is an efficient software tool that uses the latest information to functionally annotate genetic variants detected by multiple genomes [56]. Therefore, we used the package ANNOVAR (Version:2013-05-20) to annotate the SNPs.

### Principal component analysis (PCA)

In genetics, PCA is mainly applied for cluster analysis, which is based on the degree of SNP differences in individual genomes, whereby it clusters individuals into different subgroups based on the difference in their trait characteristics. PCA only targets autosomal data with an individual number n = XX, ignoring > 2 allele loci and mismatch data.

The PCA is performed as explained below:

The SNP at the k position of the individual i is represented by [0,1,2]. If the individual i is homozygous with the reference allele, then = 0; if it is heterozygous, then = 1; and if the individual i is homozygous with the non-reference allele, then = 2. M is a matrix of n × S containing the standard genotype, given below:

$$ {{\mathrm{d}}_{ik}}^{\hbox{'}}=\left({d}_{ik}-E\left({\mathrm{d}}_k\right)\right)/\sqrt{E\left({d}_k\right)\times \left(1-E\left({d}_k\right)/2\right)/2} $$

Where, E (d_k_) is the d_k_ average value, and the individual sample covariance n × n matrix is calculated by X = MMT/S. We used the GCTA [57] for PCA analysis in this calculation. To test the significance level of the Eigen vectors, we used the Tracey-Widom method.

### Linkage disequilibrium (LD) analysis

We compared the pattern of LD using high-quality SNPs. To estimate the LD decay, the degree of LD coefficient (*r*^2^) between pairwise SNPs was calculated using the Haploview software [58]. For this purpose, the parameters were set to: ‘-n -dprime-minMAF 0.05’. The average *r*^2^ value was calculated for pairwise markers in a 500-kb window across the entire genome.

### Estimation of variance components and heritabilities

In this study, the variance components and heritability were estimated using the Average Information Restricted Maximum likelihood algorithm (AI-REML) [59] in the “HIBLUP” (https://github.com/xiaolei-lab/hiblup) package of R software. The BLUP model is depicted as follows:

$$ y= Xb+ Zu+e $$

Where, y is the vector of phenotypic values; b and u represent the fixed effects and breeding values, respectively; X and Z were design matrices for b and u, respectively; e is the residual error vector with a normal distribution of e ~ N(0,Iσ_e_^2^), I is an identity matrix, and σ_e_^2^ is a residual variance. *u* ~ N(0,Gσ_*u*_^2^), in which σ_*u*_^2^ is additive genetic variance, and G is derived from genomic information and constructed by Van Raden method [26].

### Genome-wide association studies

For GWAS, the main factors resulting in false-positive associations were population structure and individual relationships [60]. EMMAX software is used for GWAS analysis which it employs a Mixed Linear Model (MLM) to correct the population structures and individual relationships (http://genetics.cs.ucla.edu/emmax/index.html). In this study, the wool traits were processed in the EMMA eXpedited (EMMAX) software. This software offers the advantage of empirical relatedness matrix containing a wide range of sample structure, including population stratification and hidden relatedness [27]. Using the general linear model to determine whether sex has an effect on phenotypic values. The threshold *p*-values for suggestive genome-wide significance was set to -log10 (*p-*value) = 7.1, *p*-value = 1/N (N is the number of SNPs for analysis). Moreover, the potential candidate SNPs were screened out by the associated significance (*p*-value) as follows:

$$ \mathrm{y}=\mathrm{X}\upbeta +\mathrm{Z}u+e $$

Where, y is an n × 1 vector of an observed phenotypic value and X is an n × q matrix of fixed effects. β is a q × 1 vector representing coefficients of the fixed effects. Z is the incidence matrix relating observations to SNP effects. u is the random polygenic effect Var(u) = σ_g_^2^ K, where K is the t × t kinship matrix inferred from genotypes, and *e* is the vector that an n × n matrix of residual effect Var(e) = σ_e_^2^ I [27].

### Identification of candidate genes and enrichment analysis

Based on the results of LD attenuation distance analysis, the biochemical function of the related genes within 50-K base pairs upstream and downstream of the physical position of the significant SNP locus were examined. The candidate genes significantly associated with the SNP loci were annotated in comparison with the sheep reference genome Ovs_aries_v4.0 (https://www.ncbi.nlm.nih.gov/assembly/GCF_000298735.2). Then, these genes were submitted to the DAVID database for Gene ontology (GO) and Kyoto Encyclopedia of Genes and Genomes (KEGG) analysis (http://david.abcc.ncifcrf.gov/).

## Supplementary Information

**Additional file 1: Table S1.** Summary of the statistics of 8 wool traits of 4 fine-wool sheep breeds.

**Additional file 2: Table S2.** The sequencing data and quality assessment of each sample.

**Additional file 3: Table S3.** 460 samples mapping and coverage statistics.

**Additional file 4: Table S4.** The linkage disequilibrium decay in populations of AMS, CMS, QHS, and AHS.

**Additional file 5: Table S5.** Summary information of significant SNPs with *p*-value<2E-06.

**Additional file 6: Table S6.** 460 phenotype data of wool traits.

**Additional file 7: Fig. S1.** SNP density across the genome for data of the four fine-wool sheep breeds.

**Additional file 8: Fig. S2.** The LD decay of the four fine-wool sheep breeds.

## Data Availability

The sequenced datasets generated and analyzed during the current study are publicly available from GenBank with the Bio project (SRA accession: PRJNA680869). The phenotype data of wool traits is provided in Additional file 6 (Table S6).

## Abbreviations

GWAS: Genome-wide association studies
SNP: Single nucleotide polymorphism
CMS: Chinese Merino Sheep
AMS: Alpine Merino Sheep
AHS: Aohan fine-wool sheep
QHS: Qinghai fine-wool sheep
FD: Fiber diameter
FDCV: FD coefficient of variance
FDSD: FD standard deviation
SL: Staple length
GFW: Greasy fleece weight
CWR: Clean wool rate
SS: Staple strength
SE: Staple elongation
QTL: Quantitative trait loci
WGS: Whole-genome sequence
PCA: Principal component analysis
LD: Linkage disequilibrium
MLM: Mixed linear model
EMMAX: EMMA eXpedited
QQ: Quantile-quantile
GO: Gene ontology
KEGG: Kyoto encyclopedia of genes and genomes
