# Supplementary figures and images for: Genome-wide association studies detects candidate genes for wool traits by re-sequencing in Chinese fine-wool sheep
Source: BMC Genomics. 2021 Feb 18;22:127. doi: 10.1186/s12864-021-07399-3 (PMC7893944; doi:10.1186/s12864-021-07399-3)

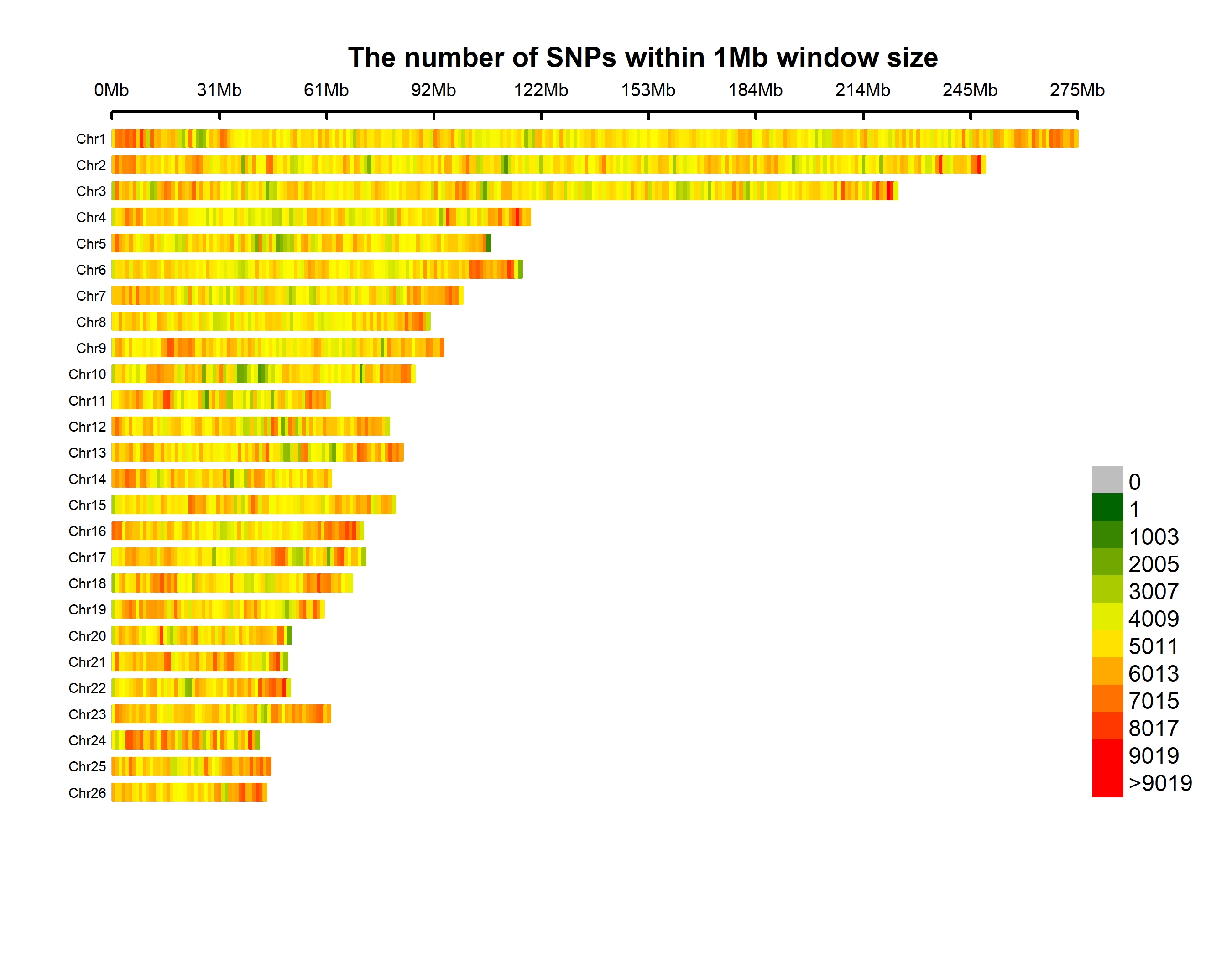

Supplement: Supplementary file 7 — Additional file 7: Fig. S1. SNP density across the genome for data of the four fine-wool sheep breeds. [file 12864_2021_7399_MOESM7_ESM.jpg]

# LD Decay

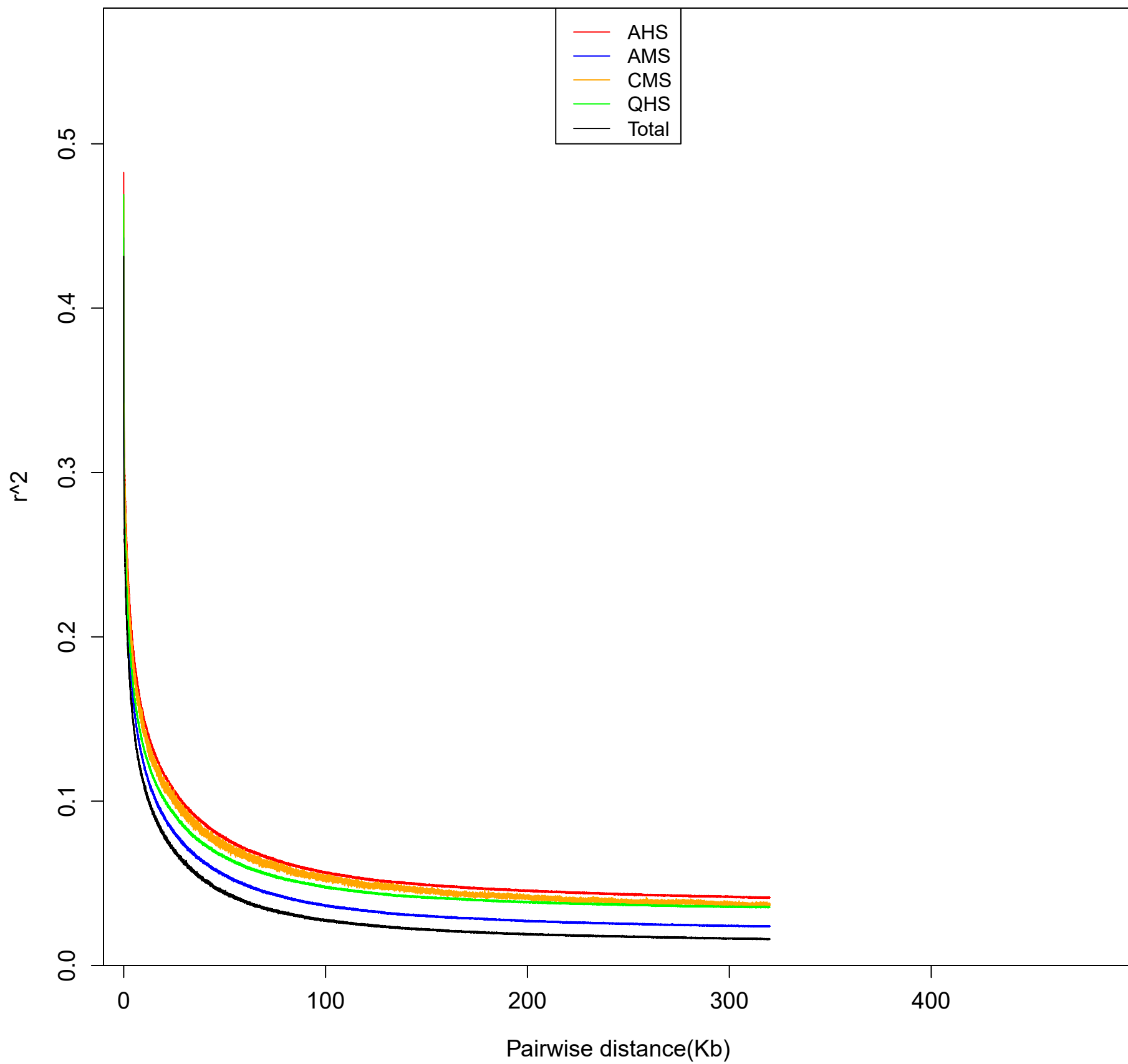

Supplement: Supplementary file 8 — Additional file 8: Fig. S2. The LD decay of the four fine-wool sheep breeds. [file 12864_2021_7399_MOESM8_ESM.pdf]
